# Spheroid Formation of Hepatocarcinoma Cells in Microwells: Experiments and Monte Carlo Simulations

**DOI:** 10.1371/journal.pone.0161915

**Published:** 2016-08-29

**Authors:** Yan Wang, Myung Hee Kim, Seyed R. Tabaei, Jae Hyeok Park, Kyuhwan Na, Seok Chung, Vladimir P. Zhdanov, Nam-Joon Cho

**Affiliations:** 1 School of Materials Science and Engineering, Nanyang Technological University, Singapore; 2 Centre for Biomimetic Sensor Science, Nanyang Technological University, Singapore; 3 School of Mechanical Engineering, Korea University, Seoul, Korea; 4 Boreskov Institute of Catalysis, Russian Academy of Sciences, Novosibirsk, Russia; 5 School of Chemical and Biomedical Engineering, Nanyang Technological University, Singapore; Lund University, SWEDEN

## Abstract

The formation of spherical aggregates during the growth of cell population has long been observed under various conditions. We observed the formation of such aggregates during proliferation of Huh-7.5 cells, a human hepatocarcinoma cell line, in a microfabricated low-adhesion microwell system (SpheroFilm; formed of mass-producible silicone elastomer) on the length scales up to 500 μm. The cell proliferation was also tracked with immunofluorescence staining of F-actin and cell proliferation marker Ki-67. Meanwhile, our complementary 3D Monte Carlo simulations, taking cell diffusion and division, cell-cell and cell-scaffold adhesion, and gravity into account, illustrate the role of these factors in the formation of spheroids. Taken together, our experimental and simulation results provide an integrative view of the process of spheroid formation for Huh-7.5 cells.

## Introduction

The study of cell culture in three-dimensional (3D) scaffolds is of considerable intrinsic interest and is also important in the context of numerous applications including, e.g., tissue engineering, disease modeling and drug screening platforms [1–3]. The structure and size of the corresponding scaffolds vary in a broad range from two-dimensional (2D) arrays of sub-millimeter wells to complex 3D structures aiming at mimicking specific organs [2, 3]. Chemically, the scaffolds are often fabricated by using natural hydrogels [2], synthetic polymers [1], or combination of such materials [4]. Cells growing in scaffolds typically aggregate. The shape and morphology of aggregates may be different, depending on various factors including the cell type, design of a scaffold and the corresponding fabrication material [1].

Cellular spheroids represent the most common shape of cell assembly [5, 6]. Aggregates of this shape were created, e.g., by concave microwell method [7], hanging drop method [5, 8], or rotating-wall vessel technique [9, 10]. The size (diameter) of spheroids may reach ~1 cm as observed in experiments with human colon adenocarcinoma cells [9] and rat hepatocytes [11] (the latter cells displayed liver-like morphology or, more specifically, a compact structure with tight cell-cell junctions, smooth and rough endoplasmic reticulum and bile canaliculi lined with the microvilli). Often, the size is smaller. For example, the size of spheroids composed of mammary epithelial cells was reported to be ~100 μm (these spheroids can produce and secrete milk proteins upon hormonal stimulation) [5], while in the case of hepatocytes the size was ~200 μm [7].

The growth of cell cultures in scaffolds is of interest also in the context of theoretical biology and statistical physics (for general introduction into this area, see reviews [12–16]). The corresponding models are usually based on the mean-field (MF) kinetic equations or Monte Carlo (MC) simulations. The MF approach is convenient in the situations where the geometry is simple. Such models were used to scrutinize the limitations in the nutrient supply and oxygen transport in porous scaffolds on the coarse-grained level without or with explicit description of single pores (see e.g. references [4, 17, 18] and [18, 19], respectively, and references therein). MC simulations, based often on the lattice approximation and describing evolution of an ensemble of individual cells, are efficient in the situations with complex geometry and/or in the cases when the focus is on aggregation of cells (as in our present study). The available generic 2D and 3D MC simulations have been focused on the growth and differentiation of stem cells [20], cell seeding [21], and formation of cell sheets [4]. Related theoretical studies concern stem-cell niches [22–25] and scaffold-less biofabrication [26].

Herein, we report the results of our study of culturing Huh-7.5 cells in microfabricated low-adhesion microwells. These cells belonging to a human hepatocarcinoma cell line are widely used as a liver cell model for the exploration of HCV infection [27]. Earlier, we observed the formation of Huh-7.5 cell spheroids in PEG-based hydrogels [28] and multilayer cell sheets in a biofunctionalized 3D scaffold [4, 29]. Our present work is focused on the same cells and has three novel ingredients.

First, we use a recently designed microwell platform for direct observation of the proliferation of cells. Its advantages include: (i) The microwell has a total depth that is two times of its diameter, and walls formed of triangular flat fragments are used to separate adjacent wells. So in contrast to conventional microfabricated semi-circular wells, this mechanical stress (shear force)-free design prevents the cells from slipping during medium exchange, and the method of fluid delivery is diffusion based. (ii) Compared to the hanging drop method [5, 8], the microwell system enables flexible medium exchange during incubation, and due to the small radius of curvature of individual wells (smaller than the size of a water drop), we are able to achieve fairly similar distribution of cell aggregates in different wells. (iii) Another feature distinguishing it from the conventional plastic round bottom wells is that the base (fabricated from silicone elastomer) is easily oxygen-permeable. The latter allows us to reduce hypoxia of cells in the centers of aggregates that is inevitable in conventional plastic scaffolds.

Second, the use of microwell platform described above allowed us to observe explicitly in detail the formation of spherical Huh-7.5 aggregates on the length scale up to 500 μm, with both light microscopy and confocal fluorescence microscopy.

Third, our experimental results are complemented by 3D Monte Carlo simulations to illustrate the role of various factors (e.g., cell diffusion, cell-surface adhesion, and gravity) in the process of cell aggregation and spheroid formation.

## Materials and Methods

### Pretreatment of 3D SpheroFilm™ for cell culture

**Fig 1:**
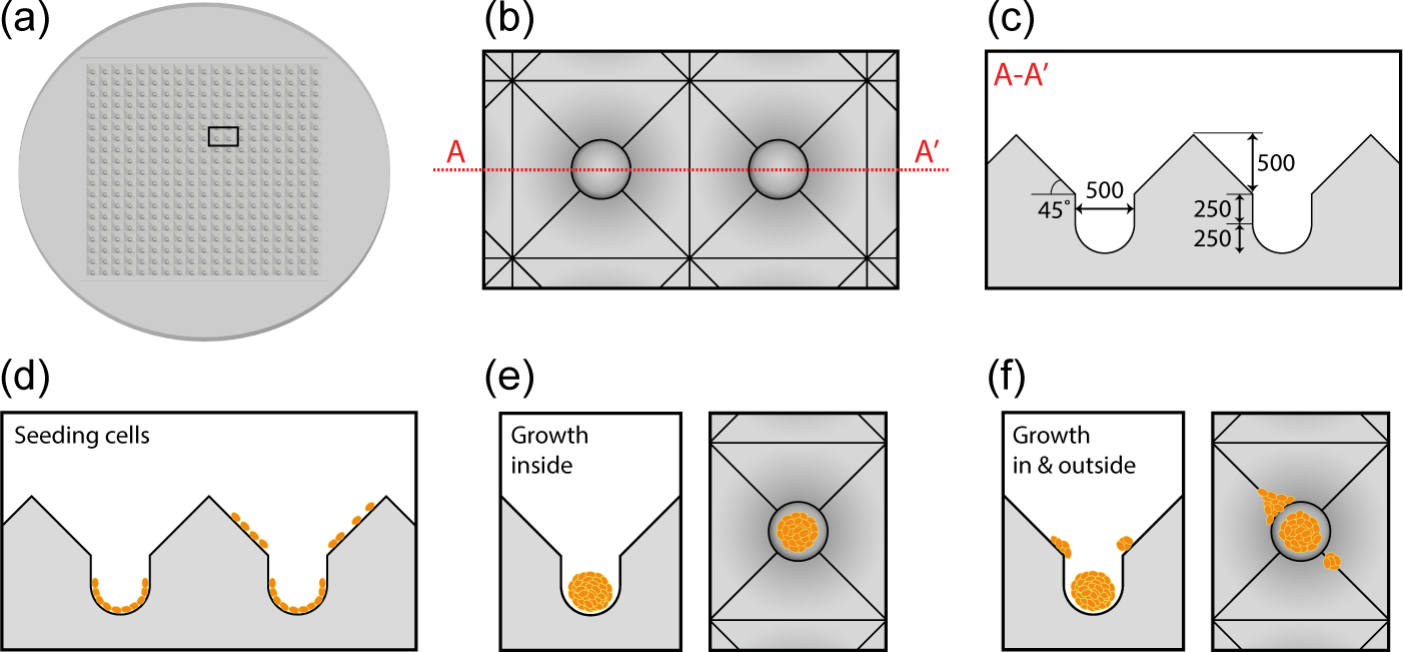
Scheme of SpheroFilm^TM^ scaffold and spheroid formation there. (a) Top view of SpheroFilm^TM^ containing 361 (19 × 19) microwells; [(b) and (c)] shape and dimensions of microwells with a diameter of 500 μm and the total well depth of 1000 μm; (d) cell seeding and attachment in and outside of microwells; (e) side and top views of spheroid formation in microwells; (f) side and top views of cell growth in and outside of a microwell.

The 3D SpheroFilm™ microwell was obtained from Incyto Co. (Chonan, Korea). The inner diameter of the hemispheres in the microwell is 500 μm, and the total well depth is 1000 μm (Fig 1). The microwell was made of mass-producible silicone elastomer. For proper cell culture, the microwell was cut to 1-cm^2^ squares, and was placed at the bottom of wells in a 24 well plate. 100% ethanol was added into the plate and repeatedly pipetted to remove the air bubbles from the wells. Once ethanol was removed, the wells were washed three times with phosphate-buffered saline (PBS), and then incubated with cell culture medium for at least 24 hours. The cell culture medium was removed prior to cell seeding.

### Cell culture and cell seeding in 3D SpheroFilm

Human hepatocarcinoma Huh-7.5 cells, purchased from Apath (NY, USA), were maintained in Dulbecco’s Modified Eagle’s Medium (DMEM) supplemented with 10% fetal bovine serum (FBS), 100 U/ml penicillin and 100 μg/ml streptomycin (Life Technologies) in a humidified atmosphere with 5% CO_2_ at 37°C. Medium was changed every three days. Cells were detached with 0.25% trypsin-EDTA solution (Life Technologies) from the tissue culture plate, counted, and adjusted to 0.05, 0.1 and 0.2 × 10^6^ cell/ml. 1 ml cell suspension was seeded in each piece of microwell square, cut and placed in 24 well plate. After 10 minutes of cell seeding, suspending cells were removed by aspiration, and the remaining cells were washed and incubated in fresh growth media until time of assay.

### Immunofluorescence staining and imaging

Cells in the microwells were collected at various stages for immunocytochemistry. Cells were washed twice with PBS, fixed with 4% paraformaldehyde (PFA) for 10 minutes, permeabilized with 0.1% Triton X-100 in PBS for 30 minutes, washed again with PBS and incubated in blocking buffer (3% bovine serum albumin (BSA) in PBS) for 1 hour. Cells were stained with mouse primary antibody against Ki-67 (Life Technologies) by overnight incubation at 4°C, and then washed three times with PBS to remove unbound primary antibody. The cells were then incubated with anti-mouse secondary antibody conjugated with Alexa Fluor® 488 (Life Technologies). Meanwhile, filamentous actin (F-actin) was stained with Alexa Fluor® 555 labelled phalloidin (Life Technologies) for 2 hours at room temperature (protected from light). After two washes with PBS, the nuclei were stained with 10 μg/ml DAPI (Life Technologies) for 30 minutes. Fluorescent cell images were taken on a LSM 710 confocal microscope with ZEN program (Carl Zeiss).

## Results and Discussion

### Cell growth and spheroid formation

**Fig 2:**
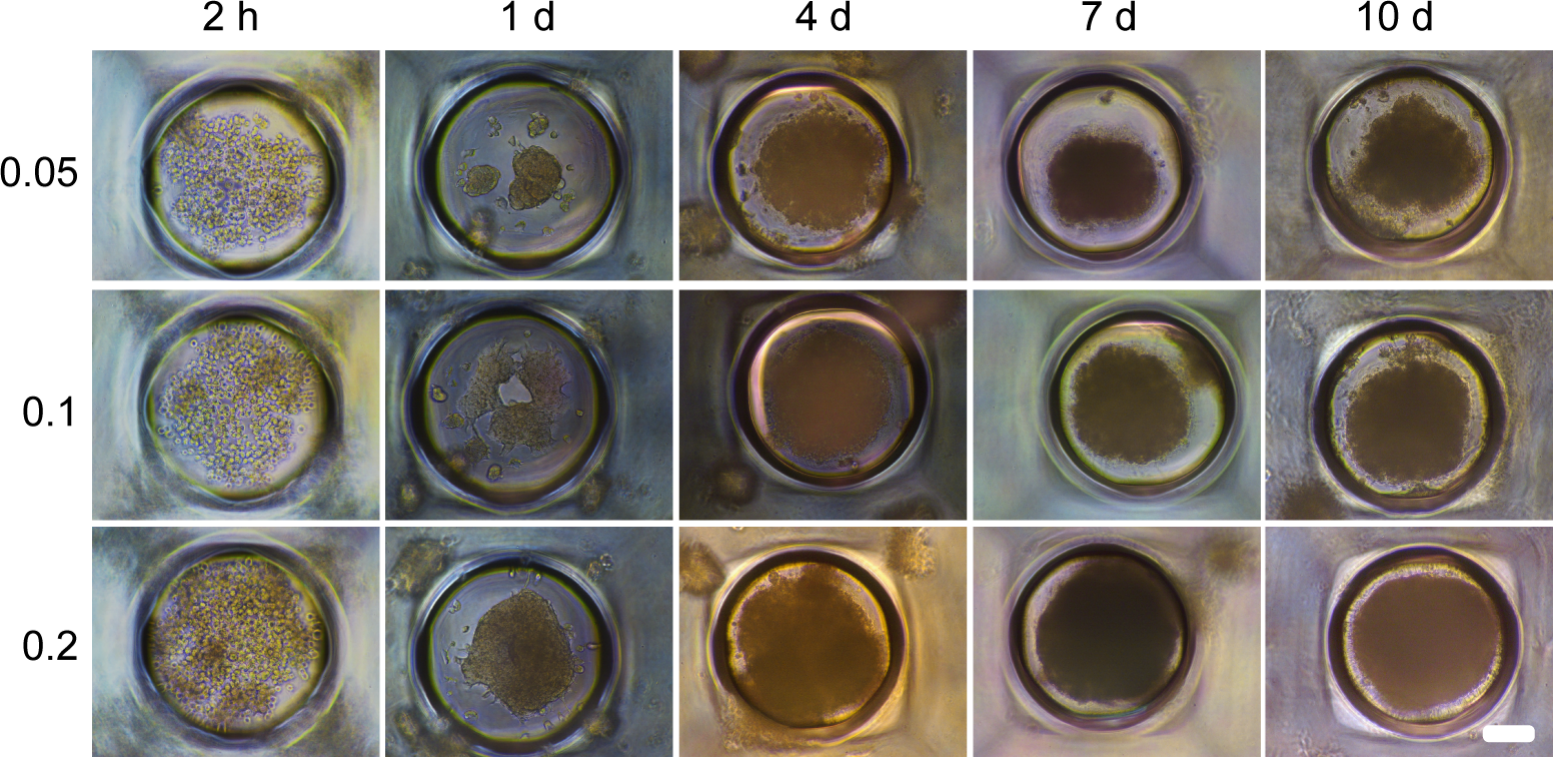
Cell growth and spheroid formation in the microwells. Three different densities of Huh-7.5 cells (0.05, 0.1 and 0.2 × 10^6^ cell/ml) were seeded into the platform and the cells were observed under light microscope over 10-day period. Scale bar is 100 μm.

The shape and dimensions of 3D SpheroFilm^TM^ microwells are schematically illustrated in Fig 1. Huh-7.5 cells were seeded in the microwells at density of 0.05 × 10^6^ cell/ml. These cells underwent several stages of morphological changes in the following 10 days (Fig 2). Initially they were attached to the bottom of the hemispheres and randomly dispersed. Later on (Fig 2, day 1), multiple cells started to form clusters due to the seemingly random cell migration and cell-cell adhesion. After day 1, several small cell clusters as well as a number of individual cells gradually merged into large cell aggregates at the center of each well of the microwell surface (Fig 2, day 4). These pre-mature spheroids kept growing bigger and denser, and finally became mature with significant thickness and clear 3D structure at day 7 and onward (Fig 2, day 7 and day 10). When Huh-7.5 cells were seeded at higher densities (0.1 and 0.2 × 10^6^ cell/ml, respectively), similar stages of morphological changes can be observed with earlier cell aggregation and denser spheroids formation (Fig 2).

**Fig 3:**
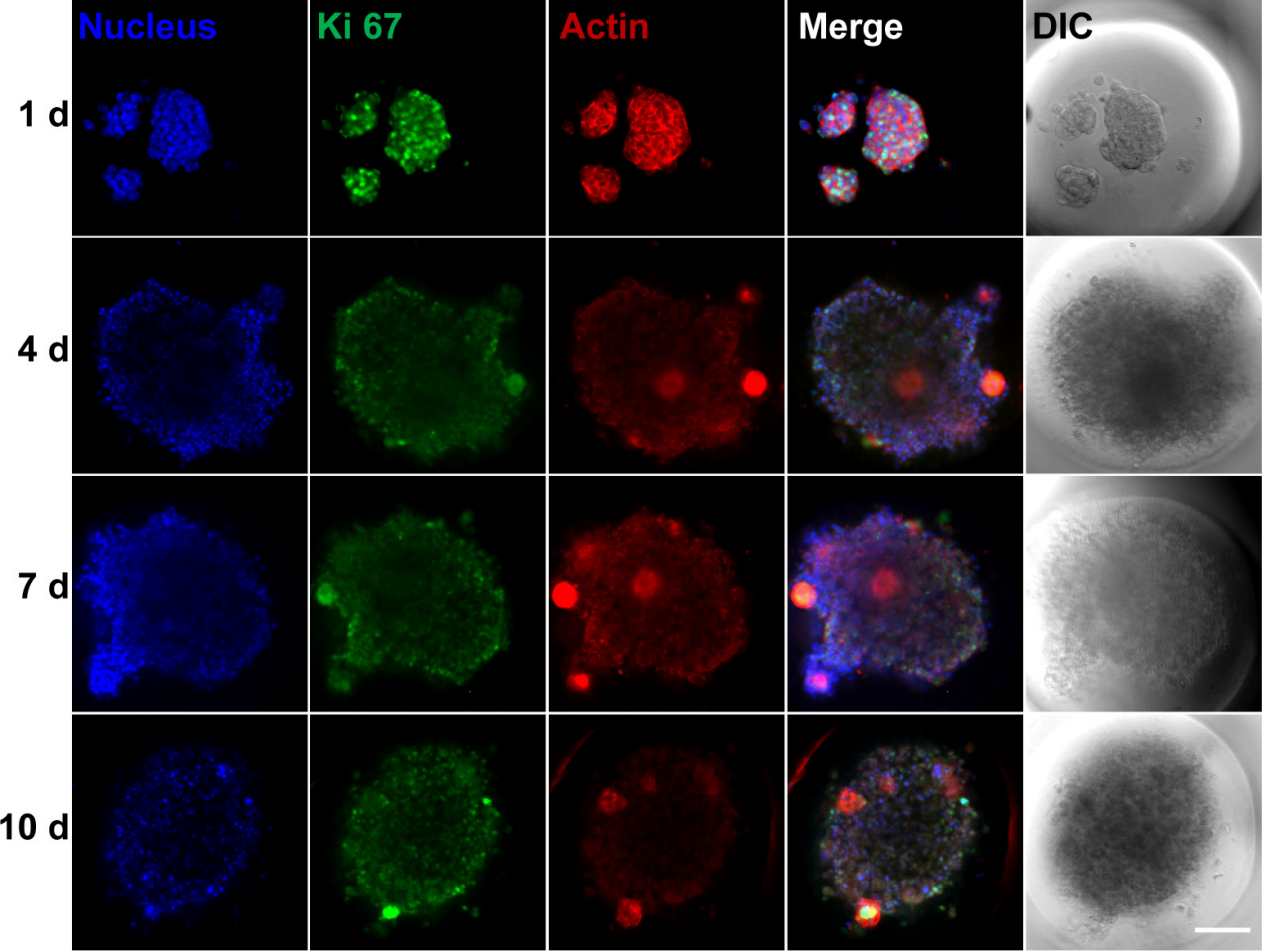
Ki-67 and F-actin staining of Huh-7.5 cells in the microwells. Huh-7.5 cells were seeded at 0.05 × 10^6^ cell/ml density, and after 1, 4, 7 or 10 days of incubation, cells were stained with DAPI for nucleus (blue), Alexa Fluor 488 for Ki-67 (green) and Alexa Fluor 555 for F-actin (red). DIC stands for differential interference contrast images. The bright green and red circles observed at days 4, 7 and 10 are cells that strongly express Ki-67 and F-actin (see white arrows). Scale bar is 100 μm.

**Fig 4:**
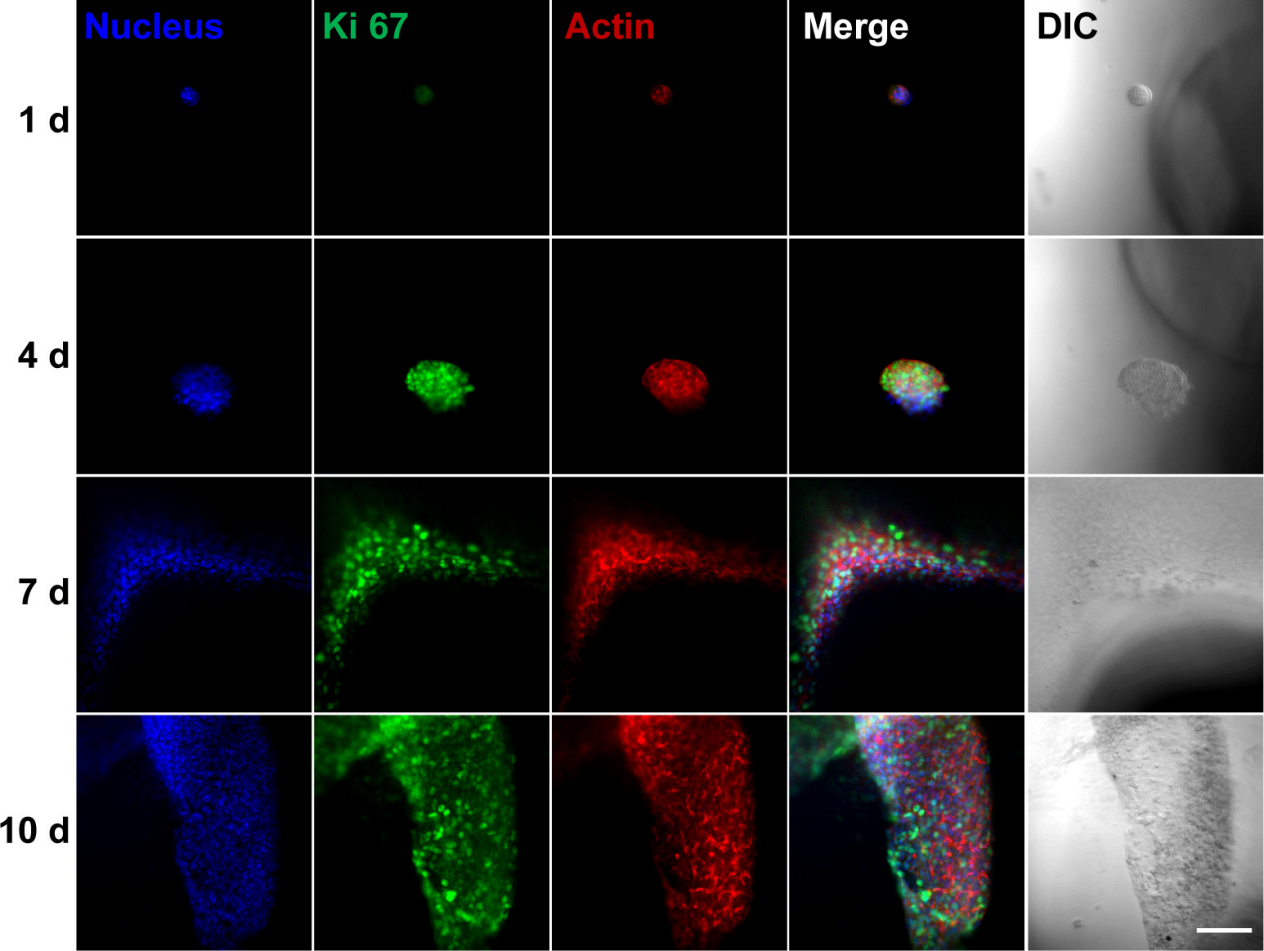
Ki-67 and F-actin staining of Huh-7.5 cells on flat surface outside the microwells. Scale bar is 100 μm.

The cell growth patterns were further examined by fluorescent staining of Ki-67 and F-actin. Ki-67 is used as a marker of proliferating cells [30–32]. The expression of Ki-67 is known to be upregulated in the G1, S, G2 and M phases of cell cycle, and reaches maximum in G2 and M phases [33, 34]. In spheroid cell culture (Fig 3), Huh-7.5 cells showed intense Ki-67 staining (green fluorescence) at day 1 after cell seeding, but the green fluorescence was markedly reduced at day 4 compared to day 1. The expression of Ki-67 at day 7 and 10 was similar to day 4. This observation indicates that the rate of cell proliferation was fastest at day 1, and it slowed down at day 4 and the rate was somewhat held constant till day 10. Another interesting observation is the spatial distribution of Ki-67 expression across the spheroid. At day 1, Ki-67 staining is evenly distributed across the cell aggregates; however, at days 4, 7 and 10, strong Ki-67 cells were found scattered on the periphery of spheroids (see white arrows), meaning that cell proliferation was globally suppressed after day 4, but some cells on the periphery still maintained their proliferation activity. Interestingly, although cells in the spheroids showed suppressed growth rate, the cells growing outside the microwells (those attached to the flat surface) demonstrated consistent high expression of Ki-67 (Fig 4).

The expression of F-actin displayed similar trend with Ki-67 (Figs 3 and 4). Concerning this aspect, we note that Chang and Hughes-Fulford earlier observed remarkable difference between actin cytoskeleton of cells in monolayers and spheroids [35]. In particular, F-actin stress fibers were found in cells of monolayer culture, while cells in spheroids featured cortical actin that clearly distributed at the outline of the cells [35]. They also reported the upregulation of structural genes including cytoskeletal molecules in monolayers. Thus, it seems to be reasonable to attribute the strong F-actin staining of cells in the microwells at day 1 (Fig 3) as well as cells outside microwells at all the time points (Fig 4) to the cell-substrate interaction and monolayer morphology, whereas when there is more cell-cell interaction in cell spheroid (Fig 3), the F-actin expression is weaker.

### Monte Carlo simulations

In reality, the proliferation of cells is usually accompanied by their aggregation due to cell-cell adhesion. In microwells, the aggregation may take place near the microwell walls due to adhesion of cells to the wall surface, and it may result in the formation of cell layers attached to the walls. The size and structure of cell aggregates depend on various factors including the adhesion strength, balance between the rates of cell division and diffusion, structure of the microwell, cell-cell communication, and limitations in the nutrient or oxygen supply, etc. Our present 3D lattice MC simulations are focused on the proliferation of cells in a single well of the microwell system (Fig 1). The emphasis is on adhesion, diffusion and the likely role of gravity in this process. The corresponding parameters are varied in a wide range in order to provide a general view on the pattern formation in the system under consideration. Some other general factors are ignored in our simulations (which is inevitable due to the complexity of the system). In particular, the cell-cell communication is not taken into account primarily because at the moment we have no data specifying this factor. Concerning this aspect, we may note that the use of the Ki-67 marker indicates that in our experiments the cell growth occurs mainly at the periphery of aggregates. It may be related to cell-cell communication and also to spatial constraints on the cell division. Our simulations take the latter factor into account, and accordingly the results are expected to be robust even if the former factor is ignored. The limitations of the nutrient or oxygen supply are not taken into account either. Such limitations are expected to be significant roughly on the aggregate length scale larger than 200–400 μm [36]. The aggregates we observe (Fig 3) are smaller than or comparable to this length scale, and accordingly the nutrient or oxygen supply is not expected to terminate the cellular growth.

To mimic the hemispherical well, we use a 60 × 60 × 80 slab of a cubic lattice. This slab is cut down to a hemisphere in the lower part (at *z* < 30, where *z* is the vertical coordinate measured in the lattice spacing units) and a cylinder in the upper part (at 30 ≤ *z* ≤ 80). Each lattice site can be either vacant or occupied by one cell. Cells can diffuse and divide. As usual in the lattice models, the diffusion of cells is realized *via* jumps of monomers to vacant nearest-neighbour (nn) sites. Diffusion (sedimentation) of aggregates is not taken into account partly due to the lack of simple suitable algorithms and partly because in our case (during the cell proliferation) this process is expected to be important only at the initial stage of the whole process (when the aggregates are small and their number is large) while we are more interested in the situation when the aggregates are relatively large.

Division of a cell is also considered to be possible provided it has a vacant nn site. After division, the cell remains on the same site and another (newly born) cell occupies a vacant nn site. Both for diffusion and division, we use the so-called no-flux boundary conditions, i.e., a jump or division is possible if an nn vacant site belongs to the array of sites representing the well.

To mimic the cell-cell and cell-surface adhesion, we introduce the effective attractive dimensionless (i.e., normalized to *k*_B_*T*) interaction, *ϵ*_cc_ < 0, between two cells located in nn sites and the effective attractive dimensionless cell-surface interaction, *ϵ*_cs_ ≤ 0, for cells contacting the boundary representing the wall of microwells. In this case, the aggregation is known to occur at |*ϵ*_cc_| > 0.89 [37]. In our calculations, we set *ϵ*_cc_ = −1.2. The value of the other interaction, *ϵ*_cs_, is varied to illustrate various situations.

To specify diffusion of monomers in the lattice models, one can use various prescriptions, e.g., those corresponding to the initial-state, Metropolis, or Kawazaki dynamics [38]. We employ the initial-state dynamics which reasonably predict rapid diffusion of single cell and slow diffusion inside aggregates. In this case, the jump rate is reduced by exp(*nϵ*_cc_) if a cell has *n* neighbours. If a cell contacts the boundary, the jump rate is reduced by the additional factor, exp(*mϵ*_cs_), where *m* is the number of the corresponding contacts. To characterize the relative rates of division and diffusion, we use the dimensionless parameter *p*_div_. The rates of division and diffusion are considered to be proportional to *p*_div_ and 1 –*p*_div_, respectively.

With the specification above, our MC algorithm consists of sequential trials to realize diffusion or division events. A site is chosen at random. If the site is vacant, the trial ends. Otherwise, a cell located in the site tries to diffuse if *ρ* > *p*_div_ or to divide if *ρ* < *p*_div_, where *ρ* (0 ≤ *ρ* ≤ 1) is a random number. In both cases, one of the nn site is selected at random and if the latter site is vacant, the division is performed with unit probability, while the diffusion jump is realized with the probability exp(*nϵ*_cc_) or exp(*nϵ*_cc_ + *mϵ*_cs_) depending on the local arrangement. After each trial, the time is incremented by |ln(*ρ*)| / *N*, where *N* is the number of lattice sites, and *ρ* (0 ≤ *ρ* ≤ 1) is another random number. With the latter prescription, the unit time is, as usual, identified with MC step (MCS). On average, one MCS corresponds to *N* MC trials. All the MC runs were started with five cells located on the lattice at random (for the representative results with rapid diffusion, the details of the initial arrangement of cells are insignificant). To characterize the cell concentration, we use the average occupation of sites, *ϑ*. The MC runs were performed up to reaching *ϑ* = 0.4.

**Fig 5:**
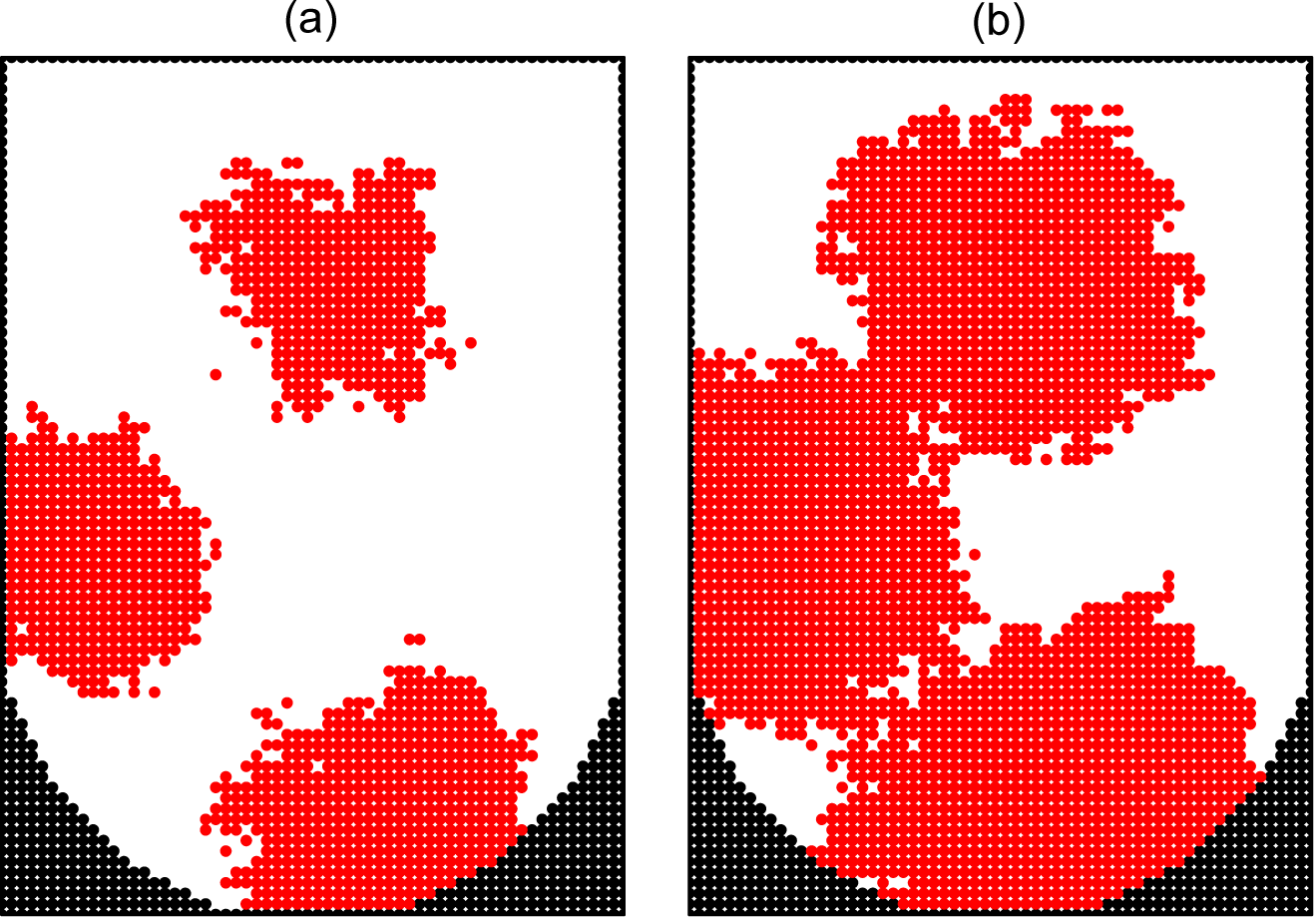
Monte Carlo simulation of cell proliferation and spheroid formation in the microwells. Cross sections of a microwell along the main axis at *ϑ* = 0.2 (a) and 0.4 (b) in the absence of cell diffusion and cell-surface adhesion (*p*_div_ = 1, *ϵ*_cs_ = 0). The well and cells are represented by black and red circles, respectively.

**Fig 6:**
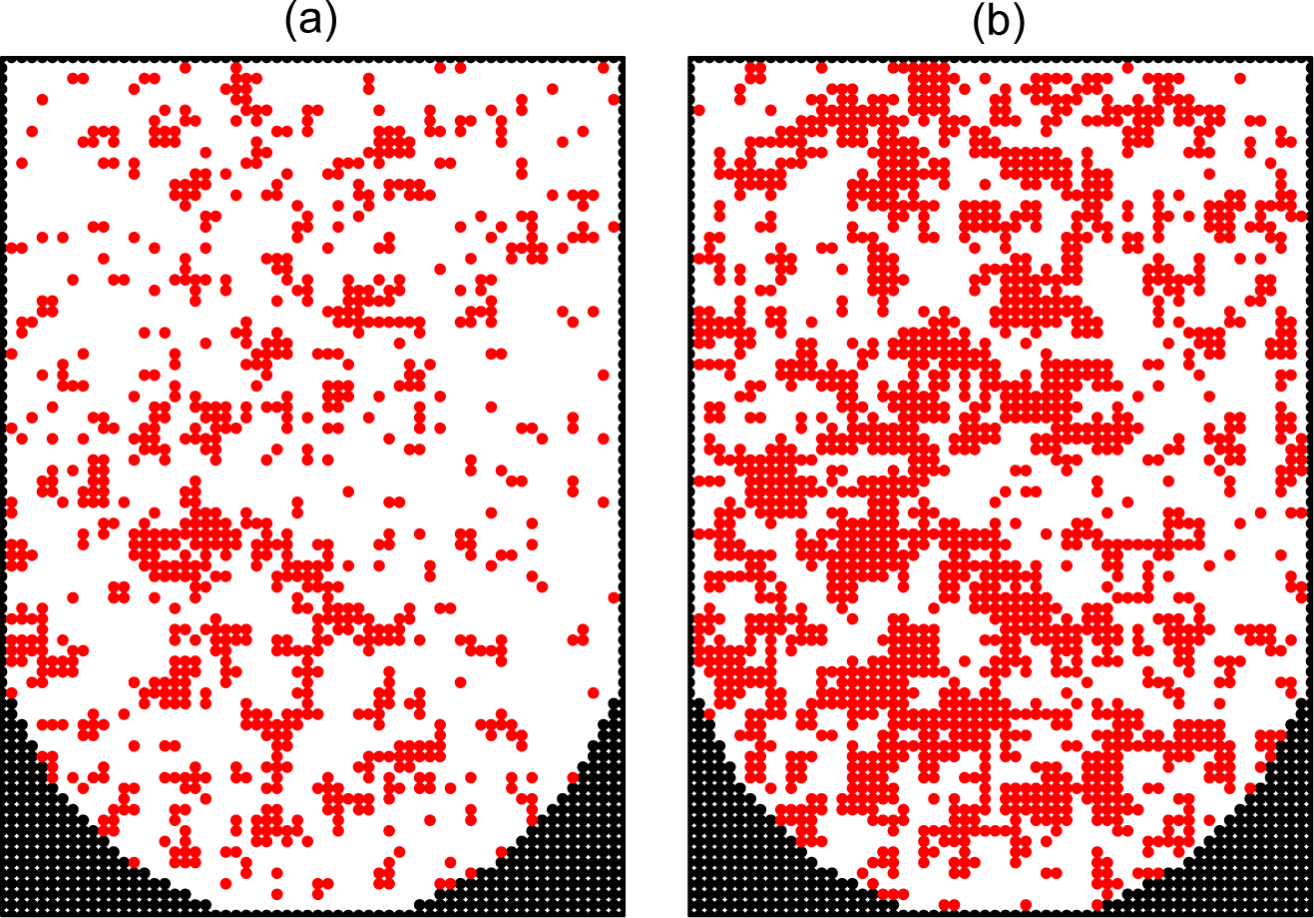
Monte Carlo simulation of cell proliferation and spheroid formation in the microwells. Cross sections of a microwell along the main axis at *ϑ* = 0.2 (a) and 0.4 (b) with slow cell diffusion (*p*_div_ = 10^−2^) and no cell-surface adhesion (*ϵ*_cs_ = 0).

**Fig 7:**
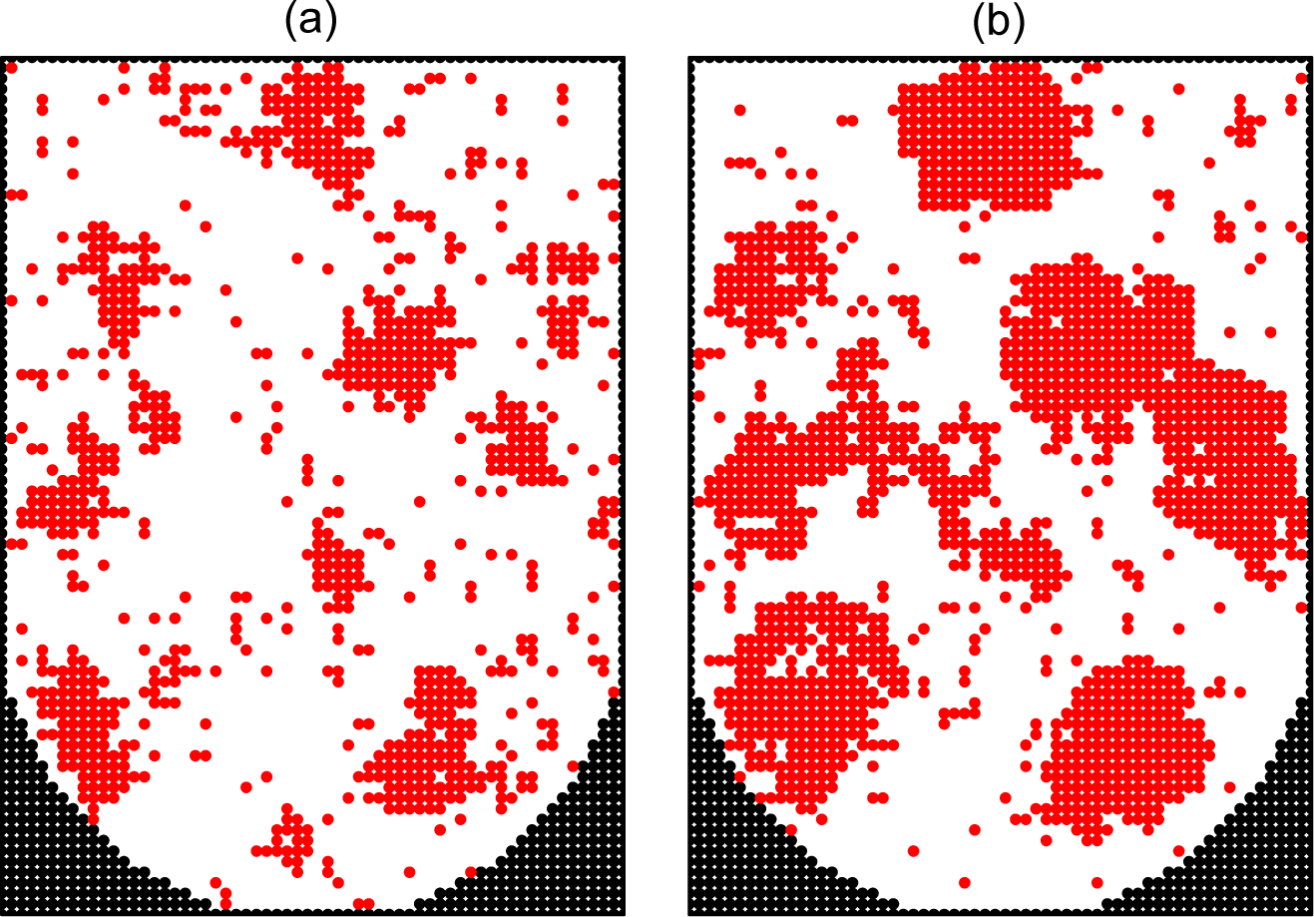
Monte Carlo simulation of cell proliferation and spheroid formation in the microwells. Cross sections of a microwell along the main axis at *ϑ* = 0.2 (a) and 0.4 (b) with relatively rapid cell diffusion (*p*_div_ = 10^−4^) and no cell-surface adhesion (*ϵ*_cs_ = 0).

In reality, the diffusion of cells is relatively rapid, and accordingly the value of *p*_div_ should be small. To get an integrated view on the process, it is instructive, however, to show typical patterns and kinetics at various rates of diffusion. Following this line, we first show patterns for *p*_div_ = 1, 10^−2^, and 10^−4^ in the absence of the cell-surface interaction, i.e. *ϵ*_cs_ = 0 (Figs 5–7). If the diffusion is negligible (*p*_div_ = 1), the growth takes place around the seed cells and there are only a few aggregates (Fig 5). In the case of slow diffusion (*p*_div_ = 10^−2^), the aggregates are numerous and their size is small (Fig 6). In the case of relatively rapid diffusion (*p*_div_ = 10^−4^), the number of aggregates becomes smaller and their size is larger (Fig 7).

**Fig 8:**
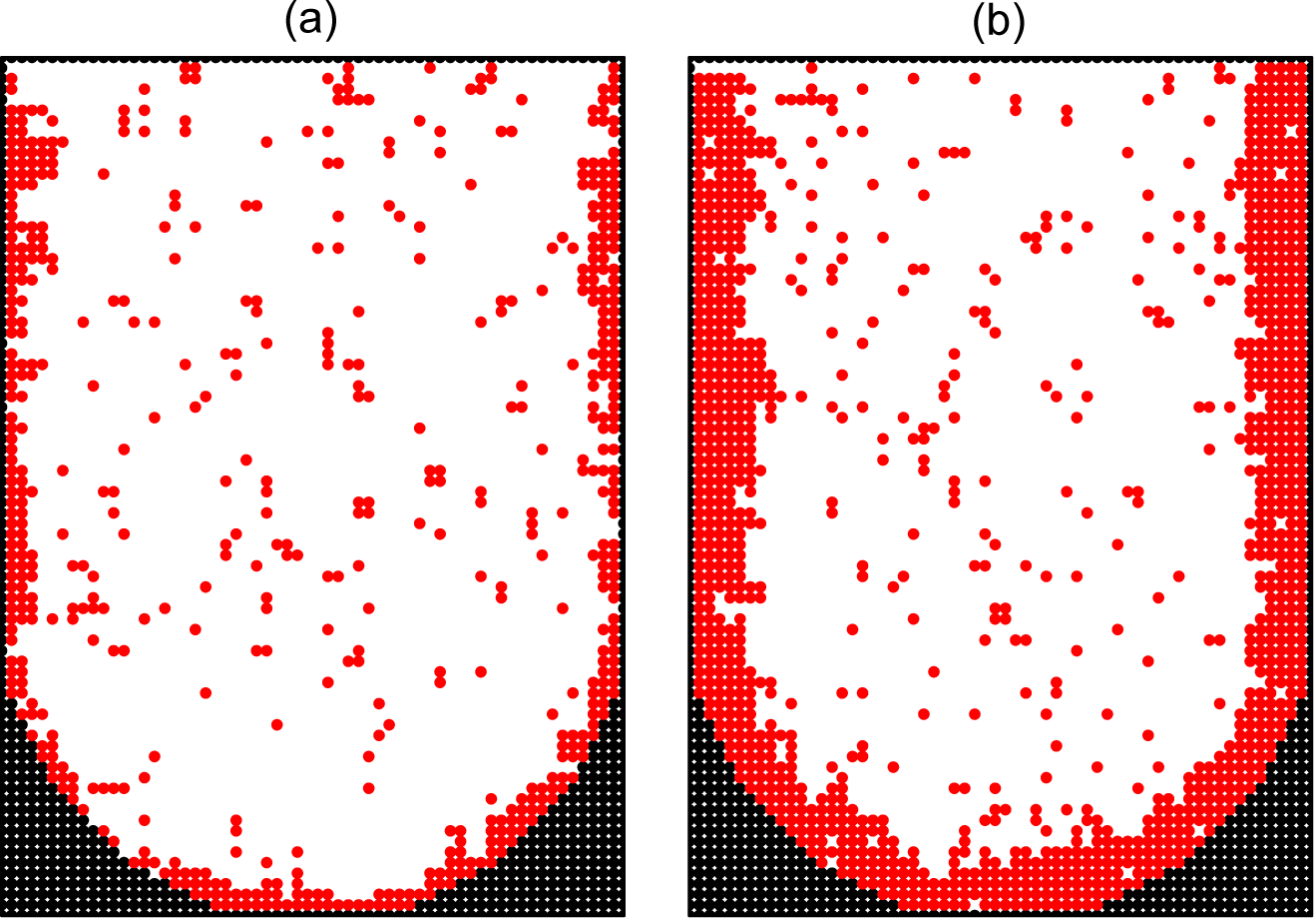
Monte Carlo simulation of cell proliferation and spheroid formation in the microwells. Cross sections of a microwell along the main axis at *ϑ* = 0.2 (a) and 0.4 (b) in the presence of adhesion (*ϵ*_cs_ = –1.2) of cells to the bottom and side area of the microwell. The adhesion to the top boundary is neglected because in reality the corresponding plane crosses the solution. The diffusion of cells is relatively rapid (*p*_div_ = 10^−4^). The well and cells are represented by black and red circles, respectively.

Secondly, using *p*_div_ = 10^−4^ and *ϵ*_cc_ = *ϵ*_cs_ = –1.2, we show the likely role of the cell-surface adhesion (Fig 8). In this case, the cell-surface adhesion is appreciable and the cells are located primarily near the walls of microwells.

**Fig 9:**
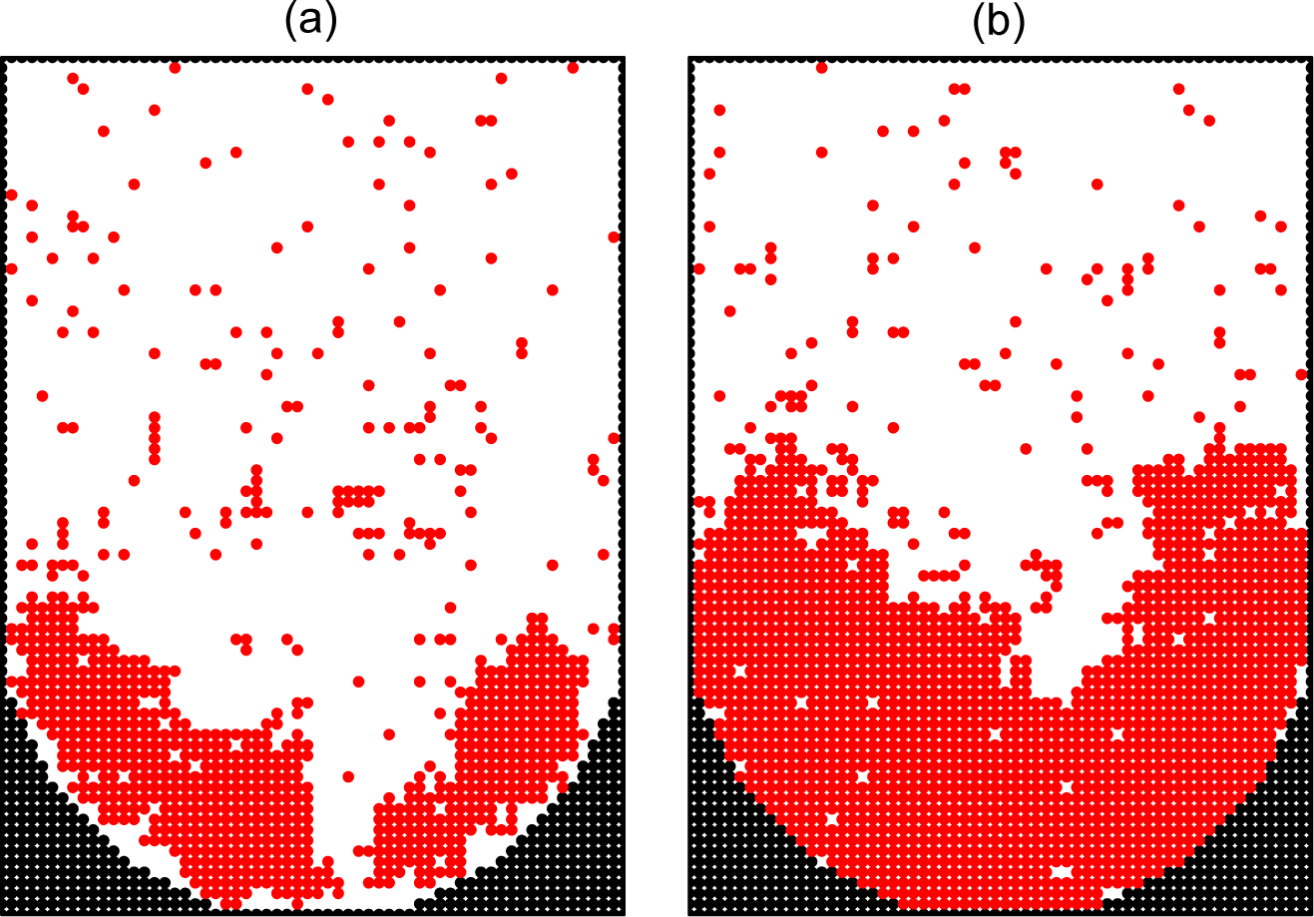
Monte Carlo simulation of cell proliferation and spheroid formation in the microwells. Cross sections of a well along the main axis at *ϑ* = 0.2 (a) and 0.4 (b) in the absence of cell-surface adhesion (*ϵ*_cs_ = 0). Cell diffusion is relatively rapid (*p*_div_ = 10^−4^). In addition, the gravity is taken into account (*ϵ*_g_ = –0.03).

In the simulations described above, the aggregates are located either at random (Figs 5–7) or near the walls (Fig 8). In our experiments, however, the cells aggregate primarily at the bottom of microwells near the center (Fig 2). This feature can be reproduced by taking the gravity into account. This factor is usually ignored in MC simulations of cell proliferation, and it was ignored in our simulations above as well. To mimic the effect of gravity, we reduce the probability of jumps upwards by a factor of exp(*ϵ*_g_), where *ϵ*_g_ < 0 is the corresponding effective energy. The results of MC simulations with *p*_div_ = 10^−4^, *ϵ*_cc_ = –1.2, *ϵ*_cs_ = 0, and *ϵ*_g_ = –0.03 show that due to the gravity the proliferation takes place near the bottom of a microwell (Fig 9).

**Fig 10:**
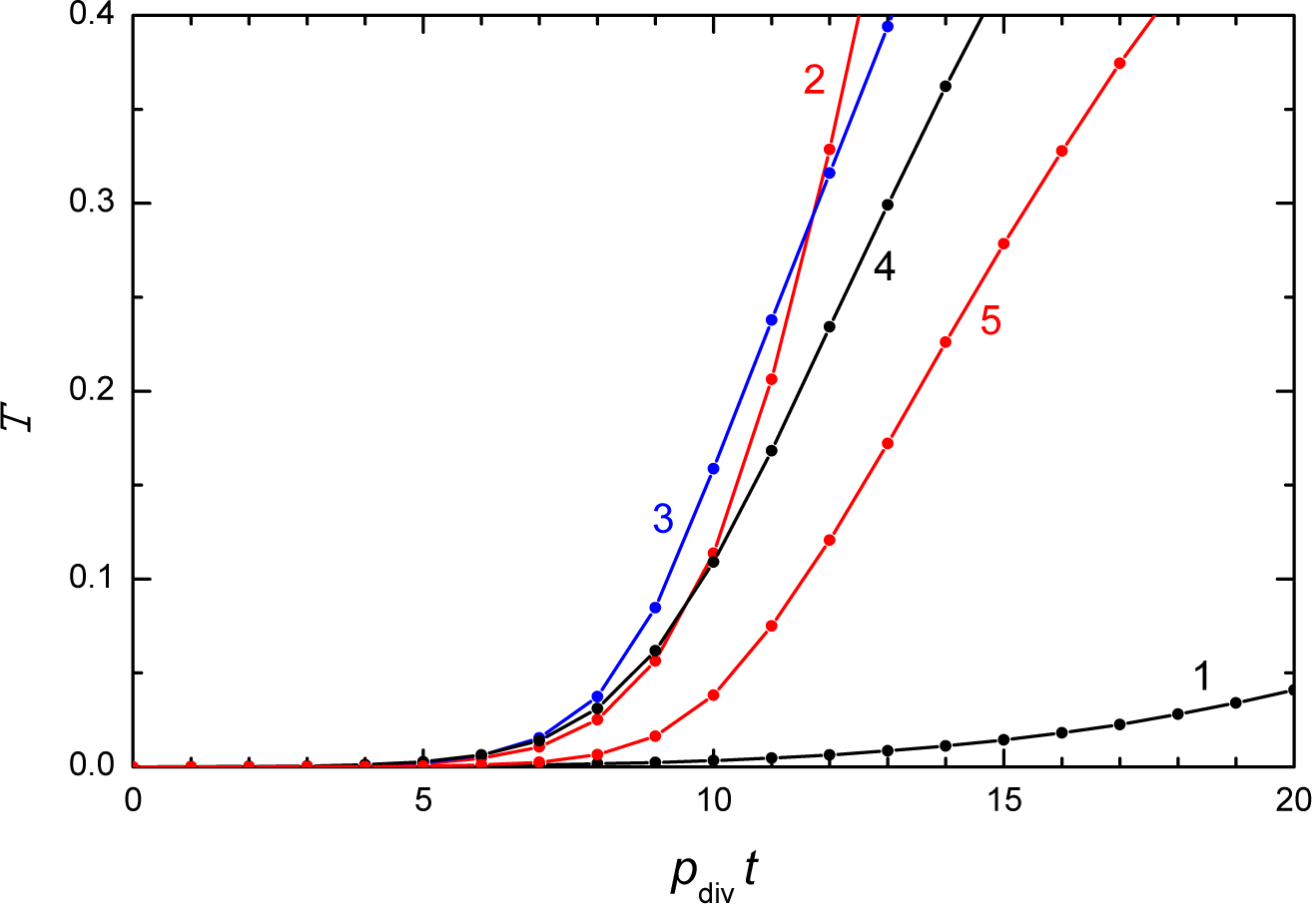
Kinetics of cell growth in the Monte Carlo simulations. Lines 1–5 represent the cell growth kinetics of MC runs shown in Figs 5–9, respectively.

Figs 5–9 exhibit typical cell growth and aggregation patterns obtained in simulations. The corresponding kinetics of cell proliferation are shown in Fig 10. In the absence of diffusion, the kinetics are slow. With diffusion, the kinetics become faster. The growth of the cell population is predicted to be exponential initially and then becomes close to linear.

## Conclusion

In this work, using a new microwell system for cell proliferation and MC simulations, we have demonstrated that the combination of experimental observation and modeling is a powerful tool for the study of cell spheroid formation. The results suggest that in the 3D cell culture platform used in our study, the cell spheroids would form on the basis of strong cell-cell interaction and weak cell-surface adhesion, and growth of spheroids mainly depends on the cell proliferation on their periphery. The likely role of the gravity in the spheroid formation was demonstrated as well. We believe that this strategy could potentially be applied to simulate even more complicated cell systems in the future. Furthermore, the capability to direct specific cells to the formation of aggregates of different types on diverse platforms, including cell spheroids in the microwells (as demonstrated in this study), cell spheroids in hydrogels of different stiffness [28], and multilayer cell sheets in biofunctionalized 3D microscaffold [4], could have significant implications for the construction and development of *in vitro* tissue models.

## References

[1] Lee J, Cuddihy MJ, Kotov NA. Three-dimensional cell culture matrices: state of the art. Tissue Eng Part B Rev. 2008;14(1):61–86. 10.1089/teb.2007.0150 18454635

[2] Varner VD, Nelson CM. Toward the directed self-assembly of engineered tissues. Annu Rev Chem Biomol Eng. 2014;5:507–26. 10.1146/annurev-chembioeng-060713-040016 24797818

[3] O'Brien CM, Holmes B, Faucett S, Zhang LG. Three-dimensional printing of nanomaterial scaffolds for complex tissue regeneration. Tissue Eng Part B Rev. 2015;21(1):103–14. 10.1089/ten.TEB.2014.0168 25084122 PMC4322091

[4] Kim MH, Kumar SK, Shirahama H, Seo J, Lee JH, Zhdanov VP, et al Biofunctionalized hydrogel microscaffolds promote 3D hepatic sheet morphology. Macromol Biosci. 2016;16(3):314–21. 10.1002/mabi.201500338 26612190

[5] Timmins NE, Harding FJ, Smart C, Brown MA, Nielsen LK. Method for the generation and cultivation of functional three-dimensional mammary constructs without exogenous extracellular matrix. Cell Tissue Res. 2005;320(1):207–10. 15714278 10.1007/s00441-004-1064-6

[6] Kim C, Chung S, Kim YE, Lee KS, Lee SH, Oh KW, et al Generation of core-shell microcapsules with three-dimensional focusing device for efficient formation of cell spheroid. Lab Chip. 2011;11(2):246–52. 10.1039/c0lc00036a 20967338

[7] Wong SF, No da Y, Choi YY, Kim DS, Chung BG, Lee SH. Concave microwell based size-controllable hepatosphere as a three-dimensional liver tissue model. Biomaterials. 2011;32(32):8087–96. 10.1016/j.biomaterials.2011.07.028 21813175

[8] Kelm JM, Timmins NE, Brown CJ, Fussenegger M, Nielsen LK. Method for generation of homogeneous multicellular tumor spheroids applicable to a wide variety of cell types. Biotechnol Bioeng. 2003;83(2):173–80. 12768623 10.1002/bit.10655

[9] Goodwin TJ, Jessup JM, Wolf DA. Morphologic differentiation of colon carcinoma cell lines HT-29 and HT-29KM in rotating-wall vessels. In Vitro Cell Dev Biol. 1992;28A(1):47–60. 1730571 10.1007/BF02631079

[10] Becker JL, Prewett TL, Spaulding GF, Goodwin TJ. Three-dimensional growth and differentiation of ovarian tumor cell line in high aspect rotating-wall vessel: morphologic and embryologic considerations. J Cell Biochem. 1993;51(3):283–9. 8501130 10.1002/jcb.240510307

[11] Brown LA, Arterburn LM, Miller AP, Cowger NL, Hartley SM, Andrews A, et al Maintenance of liver functions in rat hepatocytes cultured as spheroids in a rotating wall vessel. In Vitro Cell Dev Biol Anim. 2003;39(1–2):13–20. 12892522 10.1290/1543-706X(2003)039<0013:MOLFIR>2.0.CO;2

[12] Deisboeck TS, Wang Z, Macklin P, Cristini V. Multiscale cancer modeling. Annu Rev Biomed Eng. 2011;13:127–55. 10.1146/annurev-bioeng-071910-124729 21529163 PMC3883359

[13] Swat MH, Thomas GL, Belmonte JM, Shirinifard A, Hmeljak D, Glazier JA. Multi-scale modeling of tissues using CompuCell3D. Methods Cell Biol. 2012;110:325–66. 10.1016/B978-0-12-388403-9.00013-8 22482955 PMC3612985

[14] Urdy S. On the evolution of morphogenetic models: mechano-chemical interactions and an integrated view of cell differentiation, growth, pattern formation and morphogenesis. Biol Rev Camb Philos Soc. 2012;87(4):786–803. 10.1111/j.1469-185X.2012.00221.x 22429266

[15] Scianna M, Bell CG, Preziosi L. A review of mathematical models for the formation of vascular networks. J Theor Biol. 2013;333:174–209. 10.1016/j.jtbi.2013.04.037 23684907

[16] Wu J, Rostami MR, Tzanakakis ES. Stem cell modeling: From gene networks to cell populations. Curr Opin Chem Eng. 2013;2(1):17–25. 23914346 10.1016/j.coche.2013.01.001 PMC3727911

[17] Jeong D, Yun A, Kim J. Mathematical model and numerical simulation of the cell growth in scaffolds. Biomech Model Mechanobiol. 2012;11(5):677–88. 10.1007/s10237-011-0342-y 21830072

[18] Makhaniok A, Haranava Y, Goranov V, Panseri S, Semerikhina S, Russo A, et al In silico prediction of the cell proliferation in porous scaffold using model of effective pore. Biosystems. 2013;114(3):227–37. 10.1016/j.biosystems.2013.10.001 24141144

[19] Ehsan SM, George SC. Nonsteady state oxygen transport in engineered tissue: implications for design. Tissue Eng Part A. 2013;19(11–12):1433–42. 10.1089/ten.TEA.2012.0587 23350630 PMC3638538

[20] Zhdanov VP, Kasemo B. Simulation of the growth and differentiation of stem cells on a heterogeneous scaffold. Phys Chem Chem Phys. 2004;6(17):4347–50.

[21] Robu A, Aldea R, Munteanu O, Neagu M, Stoicu-Tivadar L, Neagu A. Computer simulations of in vitro morphogenesis. Biosystems. 2012;109(3):430–43. 10.1016/j.biosystems.2012.06.002 22732329

[22] Zhdanov VP. Simulation of proliferation and differentiation of cells in a stem-cell niche. Physica A. 2008;387(24):6126–36.

[23] Barrio RA, Romero-Arias JR, Noguez MA, Azpeitia E, Ortiz-Gutierrez E, Hernandez-Hernandez V, et al Cell Patterns Emerge from Coupled Chemical and Physical Fields with Cell Proliferation Dynamics: The Arabidopsis thaliana Root as a Study System. Plos Comput Biol. 2013;9(5):e1003026 10.1371/journal.pcbi.1003026 23658505 PMC3642054

[24] de Rooij DG, van Beek MEAB. Computer Simulation of the Rodent Spermatogonial Stem Cell Niche. Biol Reprod. 2013;88(5):131 10.1095/biolreprod.113.108639 23536371

[25] Ovadia J, Nie Q. Numerical Methods for Two-Dimensional Stem Cell Tissue Growth. J Sci Comput. 2014;58(1):149–75. 24415847 10.1007/s10915-013-9728-6 PMC3883546

[26] Sun Y, Yang XF, Wang Q. In-silico analysis on biofabricating vascular networks using kinetic Monte Carlo simulations. Biofabrication. 2014;6(1):015008 10.1088/1758-5082/6/1/015008 24429898

[27] Blight KJ, McKeating JA, Rice CM. Highly permissive cell lines for subgenomic and genomic hepatitis C virus RNA replication. J Virol. 2002;76(24):13001–14. 12438626 10.1128/JVI.76.24.13001-13014.2002 PMC136668

[28] Lee BH, Kim MH, Lee JH, Seliktar D, Cho NJ, Tan LP. Modulation of Huh7.5 spheroid formation and functionality using modified PEG-based hydrogels of different stiffness. PLoS One. 2015;10(2):e0118123 10.1371/journal.pone.0118123 25692976 PMC4333219

[29] Kim MH, Kumar SK, Shirahama H, Seo J, Lee JH, Cho N-J. Phenotypic regulation of liver cells in a biofunctionalized three-dimensional hydrogel platform. Integrative Biology. 2016;8:156–66. 10.1039/c5ib00269a 26792030

[30] Gerdes J, Schwab U, Lemke H, Stein H. Production of a mouse monoclonal antibody reactive with a human nuclear antigen associated with cell proliferation. Int J Cancer. 1983;31(1):13–20. 6339421 10.1002/ijc.2910310104

[31] Hall PA, Woods AL. Immunohistochemical markers of cellular proliferation: achievements, problems and prospects. Cell Tissue Kinet. 1990;23(6):505–22. 2276170 10.1111/j.1365-2184.1990.tb01343.x

[32] Kaita KD, Pettigrew N, Minuk GY. Hepatic regeneration in humans with various liver disease as assessed by Ki-67 staining of formalin-fixed paraffin-embedded liver tissue. Liver. 1997;17(1):13–6. 9062874 10.1111/j.1600-0676.1997.tb00772.x

[33] Gerdes J, Lemke H, Baisch H, Wacker HH, Schwab U, Stein H. Cell cycle analysis of a cell proliferation-associated human nuclear antigen defined by the monoclonal antibody Ki-67. J Immunol. 1984;133(4):1710–5. 6206131

[34] Sasaki K, Murakami T, Kawasaki M, Takahashi M. The cell cycle associated change of the Ki-67 reactive nuclear antigen expression. J Cell Physiol. 1987;133(3):579–84. 3121642 10.1002/jcp.1041330321

[35] Chang TT, Hughes-Fulford M. Monolayer and spheroid culture of human liver hepatocellular carcinoma cell line cells demonstrate distinct global gene expression patterns and functional phenotypes. Tissue Eng Part A. 2009;15(3):559–67. 10.1089/ten.tea.2007.0434 18724832 PMC6468949

[36] Lee J, Cuddihy MJ, Cater GM, Kotov NA. Engineering liver tissue spheroids with inverted colloidal crystal scaffolds. Biomaterials. 2009;30(27):4687–94. 10.1016/j.biomaterials.2009.05.024 19524294

[37] Argyrakis P, Groda YG, Bokun GS, Vikhrenko VS. Thermodynamics and diffusion of a lattice gas on a simple cubic lattice. Phys Rev E. 2001;64(6):066108. 10.1103/PhysRevE.64.066108 11736237

[38] Zhdanov VP, Kasemo B. From a pluripotent stem cell to an ensemble of differentiated cells: Elements of theoretical tissue engineering. Phys Chem Chem Phys. 2004;6(1):138–43.

